# Classification of Diseases

**Published:** 1896-04

**Authors:** 


### Classification of Diseases.

Among the particularly notable changes, differing from previous reports, is a new system of classification of diseases. It is remarked that until recently the classification of diseases and injuries pursued was based on that of the College of Physicians of England, now deemed by the Surgeon-General to be inconsistent in many respects with our present knowledge of the causation and processes of disease. He has therefore constructed a new system, in which the causes of disability are aggregated into five principal classes : (1) infectious diseases, general and local; (2) diseases of nutrition, general; (3) structural and functional diseases of organs; (4) accidents and injuries; (5) unclassified. Many of the diseases now classed as diseases of particular organs are placed among the infectious diseases, such as tonsilitis, pericarditis, endocarditis. Of the diarrhœal diseases of the old list, acute dysentery has been placed among the infectious.—*The Sanitarian.*

